# Catalytically Active SiO_2_ Aerogels Comprising Chelate Complexes of Palladium

**DOI:** 10.3390/molecules29081868

**Published:** 2024-04-19

**Authors:** Nataliya A. Sipyagina, Nikita E. Vlasenko, Alena N. Malkova, Gennady P. Kopitsa, Yulia E. Gorshkova, Sergey Yu. Kottsov, Sergey A. Lermontov

**Affiliations:** 1 Institute of Physiologically Active Compounds of Federal Research Center of Problems of Chemical Physics and Medicinal Chemistry of the Russian Academy of Sciences, 1 Severnij pr., Chernogolovka 142432, Russia; gmxten@yandex.ru (N.E.V.); amalkova81@gmail.com (A.N.M.); lermontov52@yandex.ru (S.A.L.); 2 Institute of Silicate Chemistry of Russian Academy of Sciences, 2 Adm. Makarova nab., St. Petersburg 199155, Russia; kopitsa@lns.pnpi.spb.ru; 3 Petersburg Nuclear Physics Institute Named by B.P.Konstantinov of NRC «Kurchatov Institute», 1 Orlova Roshcha, Gatchina 188300, Russia; 4 Joint Institute for Nuclear Research, 6 St. Joliot-Curie, Dubna 141980, Russia; yulia.gorshkova@jinr.ru; 5 Institute of Physics, Kazan Federal University, Kazan 420008, Russia; 6 Kurnakov Institute of General and Inorganic Chemistry of the Russian Academy of Sciences, 31 Leninsky prosp., Moscow 119991, Russia; sergey12-17@yandex.ru

**Keywords:** aerogels, palladium, chelating ligands, sol-gel, catalysis, hydrogenation

## Abstract

A series of silica-based aerogels comprising novel bifunctional chelating ligands was prepared. To produce target aerogels, two aminosilanes, namely (3-aminopropyl)trimethoxysilane (APTMS) and *N*-(2-aminoethyl)-3-aminopropyltrimethoxysilane (AEAPTMS), were acylated by natural amino acids ((*S*)-(+)-2-phenylglycine or *L*-phenylalanine), followed by gelation and supercritical drying (SCD). Lithium tetrachloropalladate was used as the metal ion source to prepare strong complexes of Pd^2+^ with amino acids covalently bonded to a silica matrix. Aerogels bearing chelate complexes retain the Pd^2+^ oxidation state after supercritical drying in CO_2_, but the Pd ion is reduced to Pd metal after SCD in isopropanol. Depending on the structure of amino complexes, Pd-containing aerogels showed catalytic activity and selectivity in the hydrogenation reactions of C=C, C≡C and C=O bonds.

## 1. Introduction

Heterogenization of catalysts combines a number of advantages, such as mechanical strength, ease of catalyst separation and the possibility of catalyst reuse. One of the important challenges in developing new heterogeneous catalysts is to reduce the noble metal content in the catalyst while maintaining its catalytic activity. Increasing the surface area of the catalyst carrier could greatly alleviate this problem. Due to this, aerogels can be an excellent carrier for heterogeneous catalysts because of their high specific surface area and porosity [1].

There are two main types of aerogels: inorganic (SiO_2_, Al_2_O_3_, TiO_2_, etc.) and organic (resorcinol-formaldehyde, agar-agar aerogels, etc.) [2,3,4,5,6]. Of these types, SiO_2_ aerogels are the most studied. Additionally, they are the easiest to modify using silanes containing organic groups covalently bonded to the silicon atom. Synthesis of SiO_2_ aerogels involves hydrolysis of precursors (silanes) to form a gel, as well as its aging and washing from impurities, followed by supercritical drying. One of the commonly used methods for producing SiO_2_ aerogel-based catalysts is the addition of catalytic particles or its precursor at the gel preparation stage. This leads to immobilization of the catalyst in the aerogel matrix. Aerogel catalysts containing metal (Pd, Ni, Cu) or metal oxides (PtO_2_, TiO_2_, Nb_2_O_5_, NiO) were used for hydrogenation [7,8], oxidation [9,10,11,12,13,14,15,16], isomerization of different classes of organic compounds and even hydrogen production via the reforming of methanol vapor [17,18,19]. A few works were devoted to the use of aerogels as superacid catalysts [20,21].

One of the most efficient ways to obtain catalysts is the synthesis of metal complexes covalently bonded to a porous solid matrix, aerogels being the best choice among other solid supports due to their high specific surface area and mesoporosity. This approach allows the synthesis of immobilized metal complexes, which brings them closer to homogeneous metal complex catalysis in terms of the efficient use of the catalytically active metal species. Furthermore, the approach presents the possibility to receive a single-atom catalytic center (formally neutral atom or ion), according to a recent single-atom center concept [22].

Suitable ligands for coordination of the metal ion in the aerogel framework are –NH_2_, –NHCH_2_CH_2_NH_2_, –CN, –CH(COMe)_2_, R_2_P-, R_2_P(O) and some other groups. In this case, the metal complex is formed at the stage of gelation or by treating the previously prepared gel with metal salts solutions. A number of works are devoted to the synthesis of metal complexes covalently bonded to the aerogel matrix [23,24,25,26,27,28,29,30,31,32]. Reactions catalyzed by this kind of catalyst include hydrogenation of C=O, C=C and C≡C bonds [25,26], amidation [27], oxidation [28,29], amination [30], hydrosilylation [31] and Heck reactions [32].

Thus, there are various types of catalytically active aerogels, including those containing amino complexes with transition metals. Nevertheless, there are still many opportunities for solving urgent problems of fine organic synthesis using aerogels. One of these problems is the synthesis of chiral compounds for use in biology and medicine. Aerogel catalysts possessing high surface area seem to be promising surface-modified systems with chiral catalytic groups. We have not found data on the use of aerogels containing chiral groups in catalysis; furthermore, xerogels (mesoporous silica) were used as carriers for optically active components [33,34,35]. Thus, salen complexes of nickel and palladium were prepared on silica gel using 3-triethoxysilylpropylisocyanate [33]. The obtained catalysts were used in the hydrogenation reaction of imines and showed low enantioselectivity. The surface of silica gel SBA-15 was modified with the product of interaction between triethoxy(3-isocyanatopropyl)silane and (1*S*, 2*S*)-1,2-diphenylethane-1,2-diamine [34]. In the presence of the obtained chiral catalyst and [Ru(p-cymene)Cl_2_]_2_, enantioselective hydrogenation of various aromatic ketones with isopropanol was carried out, leading to the synthesis of chiral alcohols in a 1–99% yield and 0–58% enantiomeric purity. SBA-15 was modified with a complex of 3-aminopropyltrimethoxysilane (APTMS), RhCl_3_ and chiral (R)-BINAP [35]. The obtained heterogeneous catalyst was used for asymmetric hydroformylation of styrene; the reaction product yield was 89%, with an enantiomeric purity of 52%.

In continuation with the above literature, complex chiral components were chosen as sources of optical activity. At the same time, natural amino acids are the most readily accessible source of optical activity. Due to the fact that they contain a chiral atom and an active amino group, we decided to use them precisely for surface modification in order to obtain catalytically active aerogels with a possible stereodifferentiating effect. Thus, in this work we obtained aerogels containing palladium complexes with new bifunctional chelating ligands based on (*S*)-(+)-2-phenylglycine and *L*-phenylalanine to use them as hydrogenation catalysts in organic synthesis. The objectives of the study were as follows: (a) to establish the possibility of using such aerogels as hydrogenation catalysts; (b) to determine their activity and selectivity towards different functional groups and (c) to find out their possible stereo differentiation activity.

## 2. Results and Discussion

**Scheme 1:**
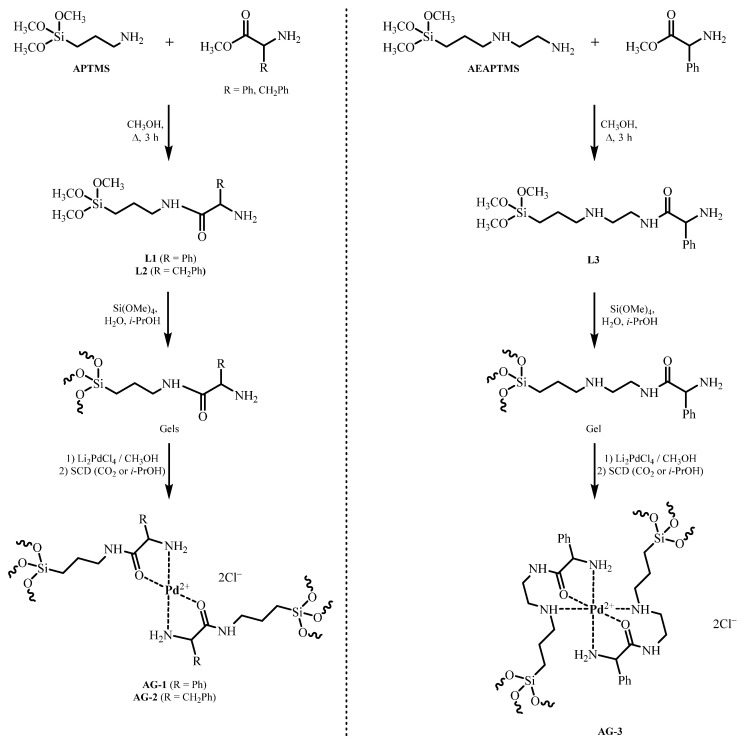
Synthesis of aerogels containing palladium amino acid complexes. APTMS—(3-aminopropyl)trimethoxysilane, AEAPTMS—N-(2-aminoethyl)-3-aminopropyltrimethoxysilane, L—Ligand, SCD—Supercritical drying, AG—Aerogel.

We have obtained palladium (II) complexes with chiral ligands on the surface of aerogels to use them as catalysts for the hydrogenation of unsaturated organic compounds with possible stereoselective action. To obtain catalysts with a chelating chiral moiety, we chose aerogels bearing (*S*)-(+)-2-phenylglycine or *L*-phenylalanine residues. We used lithium tetrachloropalladate as the metal ion source. Two aminosilanes, APTMS or AEAPTMS, were used to obtain aerogels. SiO_2_ aerogels containing novel chelated palladium complexes were prepared according to Scheme 1.

In the first step, novel bifunctional chelating ligands based on APTMS or AEAPTMS were prepared by acylation of the amino group with methyl esters of (*S*)-(+)-2-phenylglycine or *L*-phenylalanine. Since the sole acylated aminosilanes (**L1** to **L3**) proved to be incapable of forming stable gels, the chelated ligands, **L1**, **L2** or **L3,** were cogelated with tetramethoxysilane (TMOS) at a molar ratio of 1:9 to form the corresponding gels. Aerogels containing palladium ions were synthesized by sorption of Pd ions by gels from Li_2_PdCl_4_ solution in methanol. The amount of lithium tetrachloropalladate taken was based on a molar ratio of 1 mole of palladium per 2 moles of amino acid moiety. Aerogels were prepared by supercritical drying (SCD) in CO_2_, with some in supercritical (SC) isopropanol. As a result, we synthesized Pd-containing APTMS-based aerogels bearing (*S*)-(+)-2-phenylglycine and *L*-phenylalanine residues, which were designated as **AG-1** and **AG-2**, respectively. Pd-containing AEAPTMS-based aerogels carrying (*S*)-(+)-2-phenylglycine residue were designated as **AG-3**.

**Figure 1:**
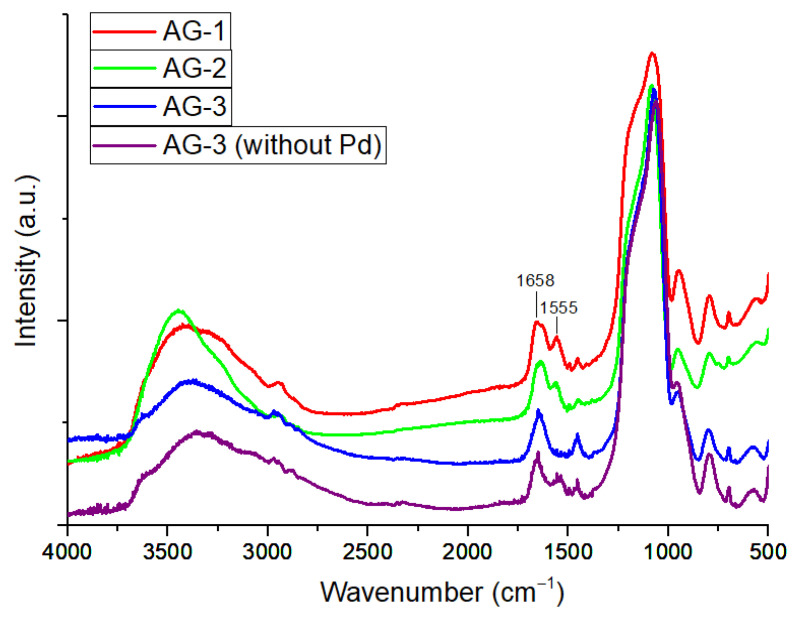
IR spectra of aerogels.

In the IR spectra of **AG-1** and **AG-2** aerogels, the absorption bands of amide I and amide II were found to be 1658 and 1555 cm^−1^ and 1637 and 1559 cm^−1^, respectively (Figure 1) [36]. The absorption band of amide I for **AG-3** was at 1643 cm^−1^. The IR spectrum of Pd-free **AG-3** aerogel is presented in Figure 1 for comparison.

**Figure 2:**
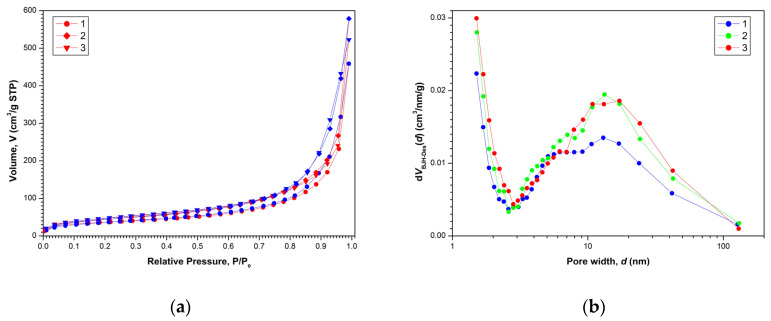
Nitrogen adsorption (red)–desorption (blue) isotherms (**a**) and pore size distributions d*V*(*d*) (**b**) for aerogel samples after SCD in CO_2_: (1)—**AG-1**, (2)—**AG-2** and (3)—**AG-3**, respectively.

The nitrogen adsorption–desorption isotherms for the aerogels are shown in Figure 2a. All presented isotherms are characterized by a certain capillary-condensation hysteresis, and according to the IUPAC classification they belong to type IV. They correspond to adsorption on mesoporous materials (containing pores from 2 to 50 nm in diameter). As can be seen in Figure 2a, the shape of the hysteresis loops for all samples corresponds to type C (according to de Boer classification), which is usually associated with the presence of open wedge-shaped pores [37].

**Figure 3:**
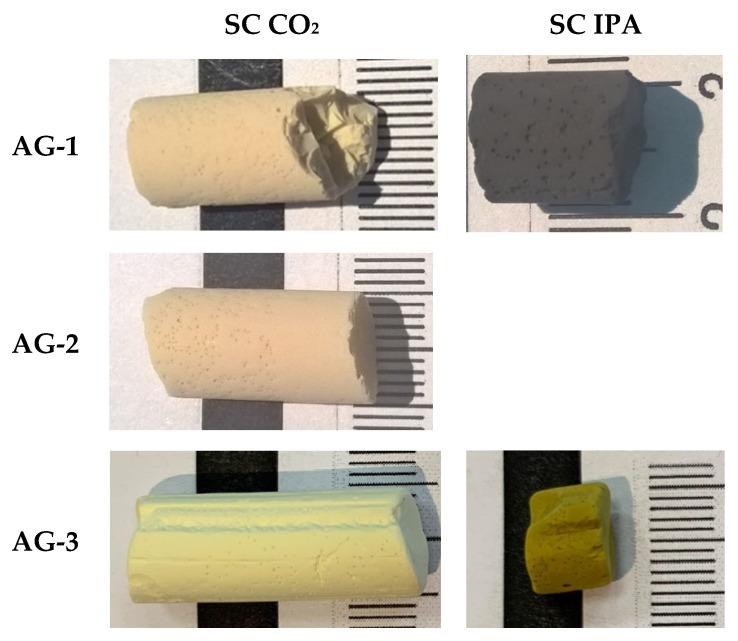
Appearance of aerogels.

Mathematical processing of nitrogen adsorption–desorption isotherms within the framework of the BJH model made it possible to determine pore size distributions d*V*(*d*) shown in Figure 2b. All aerogels exhibited the characteristic bimodal pore size distribution d*V*(*d*) with a large amount of micropores (*d* ≤ 2 nm) and a wide maximum in the *d_p_* ≈ 13 nm region. Results of the determination of texture parameters for all aerogels are presented in Table 1. Figure 3 shows the appearance of the obtained aerogels.

**Figure 4:**
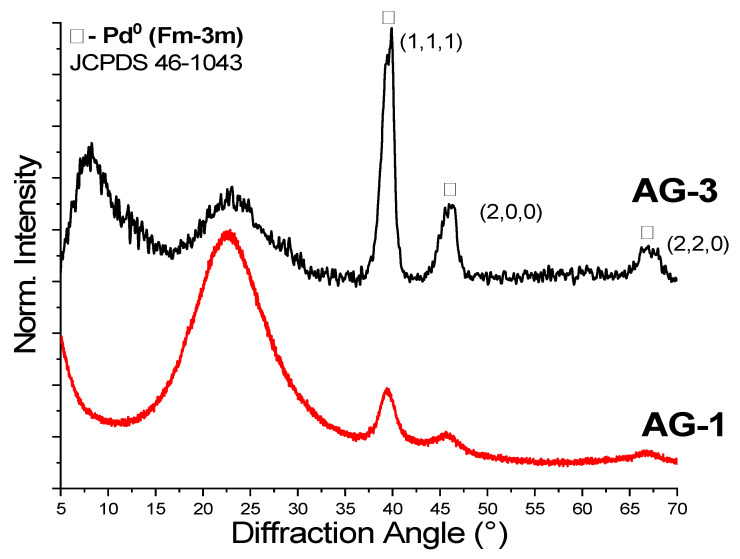
XRD of aerogel obtained after supercritical drying in isopropanol.

To study the effect of supercritical solvent on the properties of aerogels, we performed supercritical drying (SCD) in CO_2_ and in isopropanol. All aerogels dried in SC CO_2_ had a gray-yellow color, which corresponds to the color of the initial Pd^2+^ complex. A different pattern was observed for SCD in isopropanol. It was expected that the high temperature and reduction ability of isopropanol may have led to the formation of metallic palladium particles. Both **AG-1** and **AG-3** dried in SC isopropanol changed the color to dark, which is highly likely due to the presence of Pd^0^. XRD patterns (Figure 4) confirm the reduction of palladium; furthermore, diffraction peaks indexed to the crystal planes (1,1,1), (2,0,0) and (2,2,0) correspond to the cubic palladium (Fm-3m, JCPDS 46-1043), and they were observed in **AG-1** and **AG-3** aerogels. Diffraction peaks were broadened, indicating the formation of Pd^0^ nanoparticles. The crystallites’ sizes estimated by the Scherrer equation for both **AG-1** and **AG-3** were close in size, at ~5–7 nm.

**Figure 5:**
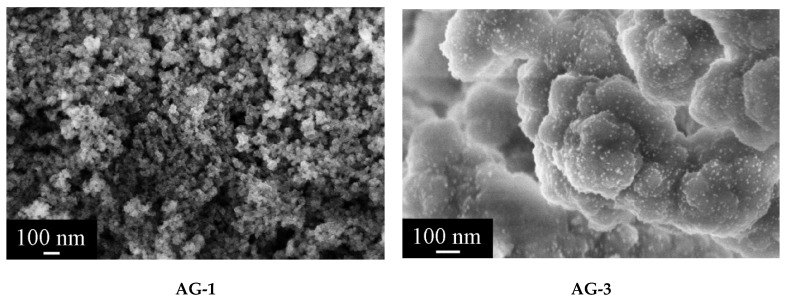
Microstructure of aerogel samples after supercritical drying in isopropanol.

According to scanning electron microscopy data, **AG-1** aerogel had a homogeneous structure after SCD in isopropanol and lacked large metallic palladium particles (Figure 5). **AG-3,** in contrast to **AG-1,** had a slightly different morphology, as the agglomerates of particles were significantly larger. Uniformly distributed particles of metallic palladium with the size not exceeding 10 nm were visible.

The reduction of Pd(+2) → Pd(0) by isopropanol needs explanation. Dehydrogenation of alcohols by Pt(0), Pd(0) and Pd(+2) compounds leading to H_2_ evolution or hydrogen transfer to C=C bonds is described in the literature [38,39,40]. It was recently shown that the palladium metal phase (PDF-2 46-1043) or the hydrogen-loaded palladium phase (PDF-2 87-0641) are formed by the reduction of Pd^+2^ species to palladium metal in supercritical isopropanol [25]. Therefore, it is obvious that metallic palladium particles are formed by the same reaction in the current study, and they serve as a true catalyst of the hydrogenation process.

**Figure 6:**
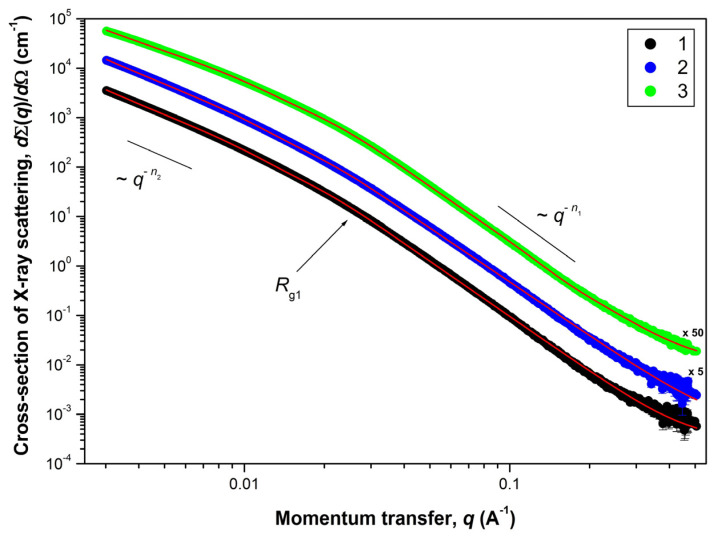
Dependencies of the Small-Angle X-ray Scattering cross-section dΣ(*q*)/dΩ versus the momentum transfer *q* for aerogel samples after SCD in CO_2_: (1)—**AG-1**, (2)—**AG-2** and (3)—**AG-3**, respectively. Solid lines correspond to the experimental data fits according to the Equation (1). For the sake of clarity, cross-section dΣ(*q*)/dΩ for samples **AG-2** and **AG-3** were multiplied by 5 and 50 (corresponding factors are given next to the curves).

Microscopy, diffraction and adsorption-based methods cannot provide comprehensive information on the organization of the supra-atomic structure in the studied aerogels. The size of individual particles (pores) and their aggregates, as well as the structure of their surface, can be obtained by applying a SAXS method, which is widely used to access the mesostructure of various materials in the 1–100 nm scale range. The experimental dependencies of the differential cross-section dΣ(*q*)/dΩ of Small-Angle X-ray Scattering on a double logarithmic scale (for aerogels obtained in SC CO_2_) are presented in Figure 6.

A common feature for all of the aerogel samples studied is the presence of three ranges in terms of the momentum transfer *q* on the corresponding scattering curves. The behavior of the scattering cross-section dΣ(*q*)/dΩ follows the power laws of *q*^−Δ^ with different values for Δ = *n*_1_ and *n*_2_, respectively. Near the crossover point *q*_c_ (the transition point from one scattering mode to another), the behavior of the scattering cross-section dΣ(*q*)/dΩ is satisfactorily described by an exponential dependence (Guinier mode). Thus, the scattering pattern observed for these aerogels is typical for scattering on two-level hierarchical porous structures [41,42,43], with a different characteristic scale and type of aggregation for each of the levels. Moreover, the convex shape of the curves dΣ(*q*)/dΩ (*n*_1_ > *n*_2_) clearly indicates that the inhomogeneities of the larger structural level are formed from the smaller inhomogeneities of the previous structural level, i.e., *R*_c2_ > *R*_c1_. Estimations of the characteristic size of inhomogeneities (*R*_c_) for each structural level can be obtained from the analysis of scattering in the Guinier mode near the crossover point *q*_c_ (in case of particles close to spherical *R*_c_ = √(5/3)∙*R*_g_ [44], where *R*_g_ is the radius of gyration of the scattering inhomogeneity). It should be noted that the estimate of *R*_c_ obtained from the analysis in the Guinier mode corresponds to the maximum size of scattering inhomogeneities of this type.

The scattering from the first (smaller in scale) structural level, observed in the region *q* > 0.03 Å^−1^, is described by the power dependence *q*^−*n*1^. The values of the exponent *n*_1_ found from the slope of the straight-line sections of the experimental curves dΣ(*q*)/dΩ plotted on a double logarithmic scale lie in the range of 3 < *n*_1_ < 4. This means that scattering takes place on inhomogeneities (pores) possessing a developed fractal surface of the phase interface (solid phase–pore) [45], the dimension of which is defined as *D*_S1_ = 6 − *n*_1_.

The scattering from the second structural level, observed at *q* ˂ 0.02 Å^−1^, is described by the power dependence *q*^−*n*2^ with exponent values of 1 < *n*_2_ < 3. It corresponds to scattering on objects (clusters) with mass-fractal aggregation of inhomogeneities [46] with fractal dimension *D*_M2_ = *n*_2_. The lower boundary of self-similarity of mass-fractal clusters of this structural level is determined by the characteristic size *R*_c1_ of inhomogeneities of the first structural level. The absence of deviation of the scattering cross-section dΣ(*q*)/dΩ dependencies from the power law *q*^−*n*2^ in the region of small momentum transfer *q* indicates that the upper boundary of self-similarity of mass-fractal clusters *R*_c2_ of the second structural level significantly exceeds the maximum size *R*_max_ that can be determined in the experiment with a given resolution. Nevertheless, using the ratio *R*_max_ ≈ 3.5/*q*_min_ [45], it is possible to make an estimation of this size, which is *R*_c2_ > 1200 Å.

Based on the above, we used the following integrated exponential-power equation for a general analysis of the observed scattering, taking into account the presence of several structural levels in the scattering system [47]:

(1) $$\frac{d\Sigma (q)}{d\Omega }=\sum_{i=1}^{2}(G_{i}\cdot \exp\left(-\frac{q^{2}R_{gi}^{2}}{3}\right)+B_{i}\exp\left(-\frac{q^{2}R_{g(i-1)}^{2}}{3}\right){\left[\frac{{\left(erf(qR_{gi}/\sqrt{6})\right)}^{3}}{q}\right]}^{n_{i}})+I_{inc}$$

The summation in Equation (1) is performed for the number of structural levels. In general, Equation (1) provides four free parameters for each structural level, such as *G_i_*, Guinier prefactor; *R_gi_*, radius of gyration; *B_i_*, power prefactor and *n_i_*, power exponent. The parameter *I_inc_* is a *q*-independent constant due to incoherent scattering on inhomogeneities close to the length of the used radiation.

To obtain the final results according to the Equation (1), the experimental dependencies of the differential SAXS scattering cross-section dΣ(*q*)/dΩ were processed using the least squares method (LSM) in the entire range under study. The results of this analysis are presented in Figure 6 and Table 2.

A comprehensive analysis of low-temperature nitrogen adsorption and SAXS data showed that aerogels samples after SCD in CO_2_ are porous two-level hierarchically organized systems with different characteristic sizes, *R*_c_ and aggregation types for each of the structural levels. The first structural level of aerogels consists of inhomogeneities (pores) with a developed fractal phase interface with fractal dimensions of 2.28 ≤ *D*_s1_ ≤ 2.38 and characteristic sizes of 225 ≤ *R*_c_ ≤ 243 Å, which at the second structural level aggregate into mass-fractal clusters with the dimension of 2.12 ≤ *D*_M2_ ≤ 2.37 and the upper limit of self-resemblance similarity of *R*_c2_ > 1200 Å. The lower limit of self-resemblance similarity of these mass-fractal clusters of this structural level is determined by the characteristic size *R*_c1_ of inhomogeneities of the first structural level.

**Scheme 2:**
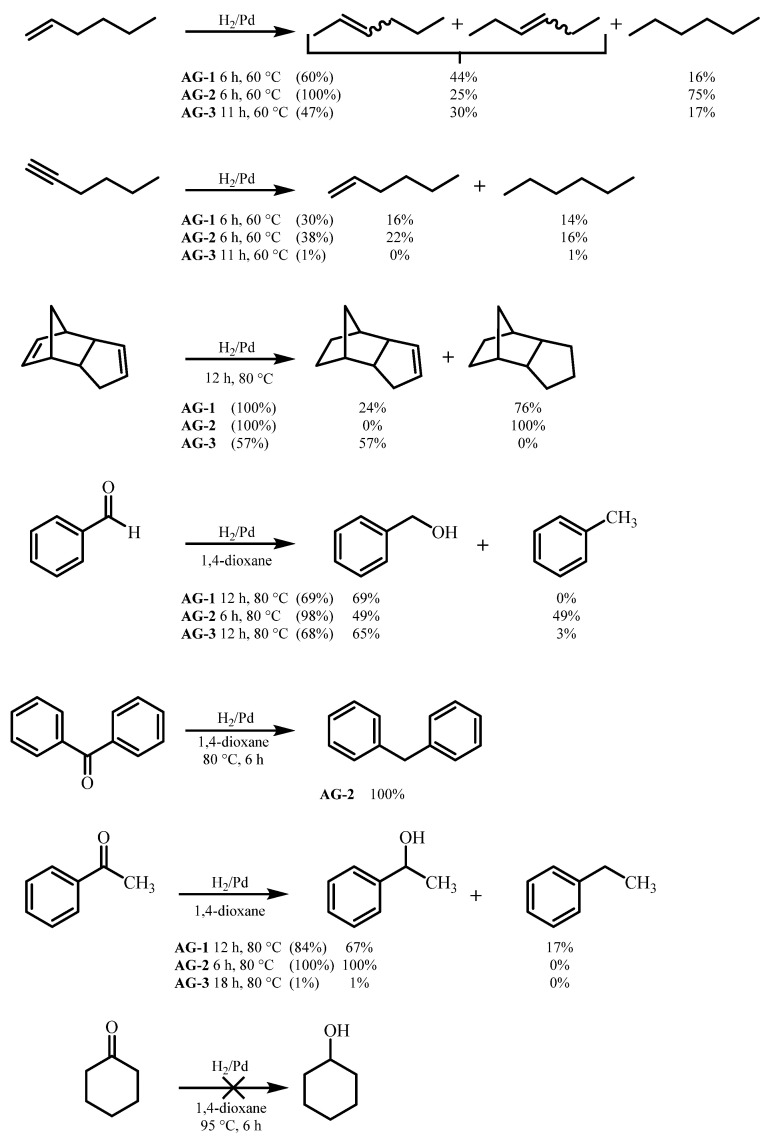
Liquid-phase hydrogenation of unsaturated organic compounds. AG—Aerogel.

CO_2_-dried **AG-1***–***AG-3** aerogels were tested as hydrogenation catalysts in model reactions of organic synthesis. The following compounds containing multiple C-C and C-O bonds were chosen as substrates: hexene-1, hexyne-1, dicyclopentadiene, acetophenone, cyclohexanone, benzaldehyde and benzophenone (Scheme 2).

The results obtained show that the synthesized complexes can be divided into two groups. The first group—**AG-1** and **AG-2** aerogels—has higher catalytic activity compared to **AG-3** in almost all hydrogenation reactions, as demonstrated by the hydrogenation of hexene-1, hexyne-1, dicyclopentadiene and acetophenone.

The lower catalytic activity of the catalyst is not always a disadvantage and is often accompanied by an increase in selectivity. This was demonstrated in the hydrogenation of dicyclopentadiene. Thus, when **AG-1** and **AG-2** were used as catalysts, the reaction products always contained complete hydrogenation products. **AG-3** allowed selective reduction of the strained norbornene double bond without affecting the cyclopentene C=C bond.

Unexpectedly, it turned out that hydrogenation of benzaldehyde leads not only to reduction to benzyl alcohol, but also to further reduction to alkane (toluene). The molar ratio of benzyl alcohol and toluene in the case of **AG-2** was 1:1. Additional confirmation of the possibility of hydrogenation of aromatic aldehydes and ketones to alkanes was the fact that benzophenone was reduced to diphenylmethane with a conversion rate of 100% on **AG-2**. Some amount of ethylbenzene was also formed during hydrogenation of acetophenone. The hydrogenation of cyclohexanone did not occur even at 95 °C.

We believe that a low activity of the **AG-3** catalyst is due to the different structure of the amino ligand. The presence of an additional amino group in **AG-3** makes it possible to form a tetra amino complex, which is usual for a Pd^2+^ ion (Scheme 1). The high affinity of palladium to amines makes this complex much stronger than in the case of C=O ligand in **AG-1** and **AG-2,** thus preventing Pd catalytic activity. We showed earlier that a similar catalyst, where palladium was chelated by aminophosphonate ligand to form a strong complex, could also be used in the hydrogenation reactions [25]. Its activity in the reduction of acetophenone was similar to that of the **AG-3** catalyst (1–2% conversion). The activity of catalysts **AG-1** and **AG-2** studied in the current work was significantly higher (84% and 100% conversion).

One of the objectives of this study was to determine the possible stereodifferentiating properties of a catalyst based on chiral amino acids. The hydrogenation of acetophenone over the **AG-2** catalyst leading to 1-phenylethanol with a potentially chiral carbon atom was chosen as a model reaction. We were able to detect only a slight excess of one of the enantiomers, and the rotation angle of the final product was 0.012° ± 0.003.

After use in the acetophenone hydrogenation reaction, the **AG-1** catalyst was separated by centrifugation, washed with methyl *tert*-butyl ether and air-dried. The palladium content in the catalyst did not change.

## 3. Materials and Methods

PdCl_2_ (Silversalt, St. Petersburg, Russia, 98%), LiCl (TTX, Sergiev Posad, Russia, 98+), isopropanol (IPA, 99.5+%, Acros, Fair Lawn, NJ, USA), tetramethylorthosilicate (TMOS, Acros, 99%), (3-aminopropyl)trimethoxysilane (APTMS, Acros, 95%), N-(2-aminoethyl)-3-(trimethoxysilyl)propylamine (AEAPTMS, Sigma-Aldrich, St. Louis, MO, USA, 97%), (*S*)-(+)-2-phenylglycine methyl ester hydrochloride (Acros, 97%), *L*-phenylalanine methyl ester hydrochloride (Acros, 98%), methanol (Acros, 99.9%), hexene-1 (Acros, 97%), benzene (Sigma-Aldrich, 99.8%), acetophenone (Acros, 98%), benzaldehyde (Acros, 98+%), benzophenone (Acros, 99%), hexyne-1 (Acros, 98%), dicyclopentadiene (Merck, Darmstadt, Germany, 98%), cyclohexanone (Acros, 99.8%) and 1,4–dioxane (Acros, 99+%) were used without further purification. Lithium tetrachloropalladate (II) was prepared by mixing 1 mol of PdCl_2_ and 2 mol of LiCl in methanol and stirring for 24 h at room temperature.

### 3.1. Preparation of Amino Acid Ligands

**2-amino-2-phenyl-*N*-(3-(trimethoxysilyl)propyl)acetamide** (**L1**): Ligand **L1** was synthesized as follows: 0.17 g (7.4 mmol) of Na was dissolved in 5 mL of methanol in a flask with reflux condenser, then 1.5 g (7.4 mmol) of (*S*)-(+)-2-phenylglycine methyl ester hydrochloride was added under stirring and heated at 50 °C for 1 h. After heating, the solution was filtered, 1.33 g (7.4 mmol) of APTMS was added to the filtrate in a flask with reflux condenser and boiled for 3 h. The resulting product was evaporated in vacuo (30 °C, 20 mbar) to remove volatile components.

^1^H NMR (CDCl_3_), δ_H_ (ppm): 0.7 m (2H, C**H_2_**–Si), 1.65 m (2H, CH_2_–C**H_2_**–CH_2_), 2.3 broad s (3H, CH–N**H_2_** и CH_2_–N**H**), 2.75 t и 3.3 m (2H, C**H_2_**–NH), 3.6 s (9H, C**H_3_**–O), 4.55 и 4.65 s (1H, C**H**), 7.4 m (5H, C_6_**H**_5_).

IR (cm^−1^): 1665 (amide I) и 1522 (amide II).

**2-amino-3-phenyl-*N*-(3-(trimethoxysilyl)propyl)propanamide** (**L2**): Ligand **L2** was obtained according to a similar methodology, using APTMS and *L*-phenylalanine methyl ester hydrochloride as precursors (see **L1** preparation).

^1^H NMR (CDCl_3_), δ_H_ (ppm): 0.6 m (2H, C**H_2_**–Si), 1.6 m (2H, CH_2_–C**H_2_**–CH_2_), 1.6 m (2H, Ph–C**H_2_**), 2.7 (1H, C**H**–CH_2_), 3.25 m (2H, C**H_2_**–NH), 3.55 s (9H, C**H_3_**–O), 7.3 m (5H, C_6_**H**_5_). ^13^C NMR (CDCl_3_), δ_C_ (ppm): 6.4 (**C**H_2_–Si), 22.8 (CH_2_–**C**H_2_–CH_2_), 41.0 и 41.4 (**C**H_2_–NH), 50.3 и 50.5 (**C**H_3_–O), 56.5 (**C**H), 126.9, 128.6, 129.2 и 138 (**C**_6_H_5_), 174.1 (**C**=O).

IR (cm^−1^): 1657 (amide I) и 1521 (amide II).

**2-amino-2-phenyl-*N*-(2-((3-(trimethoxysilyl)propyl)amino)ethyl)-acetamide** (**L3**): Ligand **L3** was obtained according to a similar methodology, using AEAPTMS and (*S*)-(+)-2-phenylglycine methyl ester hydrochloride as precursors (see **L1** preparation).

^1^H NMR (CDCl_3_), δ_H_ (ppm): 0.47–0.90 m (2H, Si–C**H**_2_), 1.43–1.75 m (2H, CH_2_–C**H**_2_–CH_2_), 2.42 broad s (4H, 2N**H** и N**H**_2_), 2.59–2.89 m (2H, C**H**_2_–NH), 3.60 s (9H, 3C**H**_3_–O), 4.53 s (1H, C**H**), 7.03–7.62 m (5H, C_6_**H**_5_).

IR (cm^−1^): 1660 (amide I) и 1520 (amide II).

### 3.2. Preparation of Aerogels

**AG-1**. 0.877 g (5.7 mmol) of TMOS and 0.2 g (0.64 mmol) of **L1** (molar ratio TMOS:**L1** = 9:1) were mixed and cooled in a plastic beaker with 1.15 g (0.0192 mol) of isopropanol. Then, 0.461 g (0.0256 mol) of cooled distilled water was added, stirred for a few seconds and gelling occurred within 30 s. The gels were then soaked with methanol for 24 h. This procedure was repeated five times.

The gels (V = 2 mL) were soaked in excess methanol solution of Li_2_PdCl_4_ (1 mL). The resulting gels containing palladium complex were soaked once daily for three days with methanol to remove unreacted Li_2_PdCl_4_ and reaction by-products. The gels were then washed with isopropanol once daily for three days. Supercritical drying in CO_2_ and isopropanol was carried out to obtain aerogels.

**AG-2** were synthesized using a similar technique (see preparation of **AG-1**). Supercritical drying in CO_2_ was carried out to obtain aerogels.

**AG-3** were prepared using a similar technique (see preparation of **AG-1**). Supercritical drying in CO_2_ was carried out to obtain aerogels.

Supercritical drying in CO_2_ was carried out in an installation composed of the Supercritical 24 constant flow pump (State College, PA, USA), a 50-mL steel reactor and GO Regulator BPR (Spartanburg, SC, USA) backpressure regulator. The aerogels samples were washed with liquid CO_2_ for 1.5 h at 20 °C and a pressure of 15 MPa, then the temperature in the reactor was elevated to 55 °C and the samples were washed with supercritical CO_2_ (15 MPa) for 2.5 h. Next, the pressure in the heated autoclave was gradually (~1 h) decreased to atmospheric pressure; the autoclave was cooled to ambient temperature and opened.

Supercritical drying in isopropanol was performed as follows: a composite gel sample in a glass tube containing 14–16 mL of isopropanol was placed into a stainless-steel autoclave (V ~ 40 mL). The autoclave was sealed and heated to the temperature of 250–260 °C (the pressure reached 6.0–7.0 MPa), exceeding the critical temperature of the isopropanol (235 °C). The heating rate was approximately 100 °C/h. After reaching the desired temperature, the valve was opened; the pressure was evenly decreased to atmospheric over 2 h. The hot autoclave was then evacuated for 30 min, cooled to room temperature and opened.

### 3.3. Characterization of Aerogels

High resolution ^1^H and ^13^C NMR spectra were obtained in CDCl_3_ using a Bruker Avance III 500 spectrometer (Bruker Daltonics GmbH, Bremen, Germany) at the Larmor precession frequencies of 500 and 126 MHz, respectively, relative to TMS.

The specific surface area *S*_sp_ of aerogels was determined by low-temperature nitrogen adsorption measurements using a Quantachrome Nova 1200e analyzer (Quantachrome Instruments, Boynton Beach, FL, USA) and a 5-point Brunauer–Emmett–Teller model (BET) within the partial pressure range of *P*/*P*_0_ = 0.05 ÷ 0.25. The specific pore volume *V*_sp_ was measured at partial pressures of nitrogen, *P*/*P*_0_ = 0.995. Pore volume distributions *D*_v_(*d*) were calculated using desorption branches of full adsorption–desorption isotherms using the Barrett–Joyner–Halenda (BJH) model. Prior to measurements, the samples were degassed at 80 °C in a dry helium flow for 17 h.

The microstructure of the samples was studied using a Carl Zeiss Supra 25 (Oberkochen, Germany) field emission scanning electron microscope (FE-SEM) at 3.96 kV acceleration voltage. Energy dispersive X-ray analysis (EDX) was performed using an Oxford Instruments INCA X-sight system (High Wycombe, Bucks, UK) operating at 12.5 kV accelerating voltage. The experiments were performed using the equipment of the Federal Research Center of Problems of Chemical Physics and Medicinal Chemistry RAS.

X-ray powder diffraction (XRD) patterns were recorded using a Bruker D8 Advance diffractometer (CuKα radiation) (Karlsruhe, Germany).

The mesostructure of the aerogels was studied using the method of Small-Angle X-ray Scattering (SAXS). The experiment on SAXS on aerogel samples was carried out using the Xeuss 3.0 SAXS/WAXS system (Joint Institute for Nuclear Research, Dubna, Russia). The radiation was generated by a microfocus X-ray source GeniX3D (CuKa, λ = 1.54 Ǻ), operating in the 30 W/30 μm mode. The beam was focused using FOX 2D optics (St. Wayland, MO, USA) and collimated using two sets of slits. The spectrometer was equipped with a moving detector Eiger2 R 1M (DECTRIS Ltd., Baden, Switzerland), with a sensitive area of 77.1 × 79.7 mm^2^ (pixel size 75 microns). The use of two distances from the sample to the detector (400 mm and 4500 mm) made it possible to measure the X-ray scattering intensity I(*q*) in the momentum transfer range of 3 × 10^−3^ < *q* < 0.5 Å^−1^.

Aerogels were measured at room temperature in a vacuum. To find the differential cross-section for Small-Angle Scattering dΣ(*q*)/dΩ in absolute units, the common procedure for normalizing the scattering cross-section of amorphous (glassy) carbon was used, which provided a plateau with an intensity of 3.805 cm^–1^ upon scattering at small angles in the range of 1 × 10^–2^ < *q* < 9 × 10^–2^ Å^–1^. The revealed value is about 250 times higher than the corresponding value for H_2_O, thus providing a more precise value for absolute scattering intensity.

### 3.4. The Reactions of Catalytic Hydrogenation

Liquid-phase hydrogenation: A flask with a thermostatic jacket was charged with 3 mL of a solvent (dioxane), 30 mg of an aerogel and 200 μL or 200 mg of the test substance (benzene, acetophenone, benzaldehyde, benzophenone, cyclohexanone). In the case of hexene-1, hexyne-1 and dicyclopentadiene hydrogenation, 3 mL of the test substance was used and no solvent was added. The installation was purged with hydrogen for 1 h, then heating and stirring with a magnetic stirrer continued for 6 h. The product solution was separated from the catalyst by centrifugation and in the case of acetophenone, benzaldehyde, benzophenone and cyclohexanone were evaporated in vacuo (30 °C, 20 mbar) to remove the solvent. The analysis was carried out using the NMR method.

## 4. Conclusions

The approach we used ensures strong binding of the palladium (+2) to the SiO_2_ aerogel matrix bearing amino acids moieties, and prevents palladium leaching in the case of supercritical drying (SCD). Aerogels containing Pd^2+^ ions retain the Pd oxidation state during drying in CO_2_ and are reduced to the metal in SC isopropanol. It was found that aerogels after SCD in CO_2_ consist of surface-fractal inhomogeneities with fractal dimensions of 2.28 ≤ *D*_s1_ ≤ 2.38 and characteristic sizes of 225 ≤ *R*_c_ ≤ 243 Å, from which, at the next structural level, mass-fractal clusters with the dimensions of 2.12 ≤ *D*_M2_ ≤ 2.37 and the upper limit of self-resemblance similarity *R*_c2_ > 1200 Å are formed.

Aerogels containing Pd ions chelated by amino acids ligands were tested as catalysts for hydrogenation of C=C, C≡C and C=O bonds of various organic compounds. The catalysts **AG-1** and **AG-2** showed high catalytic activity in the hydrogenation of C=C and C≡C bonds of the aliphatic series, with the C=O bond in aromatic aldehydes and ketones. The catalytic activity of **AG-3** in hydrogenation reactions was found to be lower than that of **AG-1** and **AG-2**. The **AG-2** catalyst was found to have a small degree of stereoselectivity in the reaction of acetophenone reduction to 1-phenylethanol.

## Figures and Tables

**Table 1:** Texture parameters of aerogel samples after SCD in CO_2_.

Sample	AG-1	AG-2	AG-3
*S*_sp_ (m^2^/g)	129 ± 2	160 ± 3	168 ± 3
*V_P_*_/*P*0≅0.995_ (cm^3^/g)	0.70	0.90	0.81
*d_p_* (nm) BJH (des)	13	13	13

**Table 2:** Mesostructure parameters of aerogel samples after SCD in CO_2_ obtained from SAXS data analysis.

Parameters	Aerogels		
	AG-1	AG-2	AG-3
*R*_c2_, Å	>1200	>1200	>1200
*B*_2_·10^3^, cm^−1^Å^−*n*2^	2.7 ± 0.2	3.4 ± 0.3	1.8 ± 0.1
*D*_M2_ = *n*_2_	2.37 ± 0.01	2.35 ± 0.01	2.12 ± 0.01
*G*_1_, cm^−1^	340 ± 20	370 ± 20	390 ± 30
*R*_c1_*=* √(5/3)∙*R*_g1_, Å	243 ± 15	240 ± 15	225 ± 15
*B*_1_·10^5^, cm^−1^Å^−*n*1^	1.40 ± 0.05	2.20 ± 0.07	2.30 ± 0.07
*D*_s1_ = 6 − *n*_1_	2.29 ± 0.01	2.38 ± 0.01	2.28 ± 0.01
*I*_inc_·10^3^, cm^−1^	0.3 ± 0.01	0.2 ± 0.01	0.5 ± 0.02

## Data Availability

Data are contained within the article.
